# Optimising Homing Endonuclease Gene Drive Performance in a Semi-Refractory Species: The *Drosophila melanogaster* Experience

**DOI:** 10.1371/journal.pone.0054130

**Published:** 2013-01-18

**Authors:** Yuk-Sang Chan, David S. Huen, Ruth Glauert, Eleanor Whiteway, Steven Russell

**Affiliations:** 1 Dept. of Genetics, University of Cambridge, Cambridge, Cambridgeshire, United Kingdom; 2 Cambridge Systems Biology Centre, Cambridge, Cambridgeshire, United Kingdom; National Cancer Institute, United States of America

## Abstract

Homing endonuclease gene (HEG) drive is a promising insect population control technique that employs meganucleases to impair the fitness of pest populations. Our previous studies showed that HEG drive was more difficult to achieve in *Drosophila melanogaster* than *Anopheles gambiae* and we therefore investigated ways of improving homing performance in *Drosophila*. We show that homing in *Drosophila* responds to increased expression of HEGs specifically during the spermatogonia stage and this could be achieved through improved construct design. We found that 3′-UTR choice was important to maximise expression levels, with HEG activity increasing as we employed *Hsp70, SV40, vasa* and *βTub56D* derived UTRs. We also searched for spermatogonium-specific promoters and found that the *Rcd-1r* promoter was able to drive specific expression at this stage. Since Rcd-1 is a regulator of differentiation in other species, it suggests that *Rcd-1r* may serve a similar role during spermatogonial differentiation in *Drosophila*. Contrary to expectations, a fragment containing the entire region between the *TBPH* gene and the *bgcn* translational start drove strong HEG expression only during late spermatogenesis rather than in the germline stem cells and spermatogonia as expected. We also observed that the fraction of targets undergoing homing was temperature-sensitive, falling nearly four-fold when the temperature was lowered to 18°C. Taken together, this study demonstrates how a few simple measures can lead to substantial improvements in the HEG-based gene drive strategy and reinforce the idea that the HEG approach may be widely applicable to a variety of insect control programs.

## Introduction

Some arthropods pose serious threats to human and animal health as well as to agriculture. Such threats may be direct, as in the case of agricultural pests, or indirect, as with vectors for disease-causing organisms. Because currently deployed approaches appear to have been ineffective in controlling some arthropods, genetically-based approaches have been increasingly investigated as an alternative route to the control or eradication of arthropod threats [1].

The homing endonuclease (HEG) gene drive system is one proposed genetic strategy [2]. Homing endonucleases differ functionally from the more well-known restriction endonucleases in that they possess longer recognition sequences of 18–22 base pairs in length. When a HEG is integrated into its recognition sequence in the genome, its protein product acts to cleave its cognate site on the homologous chromosome and gene conversion or homologous recombination can result in a new copy of the HEG being inserted. Techniques for engineering HEG target specificity have recently been developed for gene therapy [3], [4]. Burt proposed that such methods could be applied to engineer HEGs that recognise and cleave sequences within coding sequences of genes in insect genomes, with the subsequent invasion of these HEGs into a population leading to the inactivation of target genes and the subsequent decline in fitness of the targeted population [2]. In particular, HEG gene drive could be particularly effective if activity was restricted to the male germline to target genes required for female fertility/viability or engineered to destroy the X-chromosome by cutting at multiple X-specific sites [5], [6].

Natural homing endonucleases are restricted to fungal genomes and have not been identified in any metazoans to date, thus it is possible that metazoans are inherently refractory to HEG spread. Recently, the spread of HEGs *in vivo* has been demonstrated experimentally in both *Anopheles* and *Drosophila* using the model HEG, I-*Sce*I [7], [8]. However, the ease with which efficient homing was achieved in *Anopheles* was in sharp contrast to the difficulty in establishing homing in *Drosophila*. In particular, the homologous recombination activity in the *Drosophila* testis necessary for efficient homing was shown to be restricted to the spermatogonia [8]. In this paper, we describe how improvements in homing performance, on which the HEG gene drive depends, can be achieved. We also investigated the role of 3′-UTR choice, the use of spermatogonially-directed promoters, and the relationship between homing and HEG activity. We investigated factors that could potentially influence HEG drive performance, including genome context and ambient temperature and show that the latter, but not the former, has a strong influence on gene drive performance. While we initially developed the HEG system in *Drosophila* as a model for its use in the malaria mosquito, the increasing importance of controlling more closely related pest species such as *Drosophila suzukii* or the Mediterranean fruit fly *Ceratitis capitata*, suggest the development of more efficient HEG-based homing strategies could be more widely applicable in pest control [9], [10].

## Methods

### Constructs

All genomic coordinates are from Flybase Release 5.46 [11].

Only constructs novel to this work are described here. Earlier constructs are described in [8].

Promoter fragments were chosen such that they extended to and abutted the start codon with the intent of including any upstream translational-regulatory sequences that may modulate expression. The *bgcn* promoter used was an 817 bp fragment extending upstream of the start codon (2R:19747036.19746220). The *Rcd1-r* (CG9573) promoter was a 937 bp fragment extending upstream of the start codon (2L:9014859.9013923). The *bam* 3′-UTR was a 545 bp fragment extending from *bam* into the neighbouring overlapping 3′-end of the *CG11854* transcribed region (3R:21069230.21068686). The *vas* 3′-UTR was a 318 bp fragment extending across the stop codon and beyond the end of the transcribed region (2L:15074153.15074470). The *bgcn* 3′-UTR used was a 387 bp fragment spanning the entire *bgcn* 3′-UTR and part of the *gbb* 3′-UTR (2R:19741086.19740700).

The *Rcd-1r*-K227M*-βTub56D* nickase construct was created by mutating I-*Sce*I K227codon in *Rcd-1r*-HEG-2*-βTub56D* to encode methionine instead.

### Homing Assay

**Figure 1:**
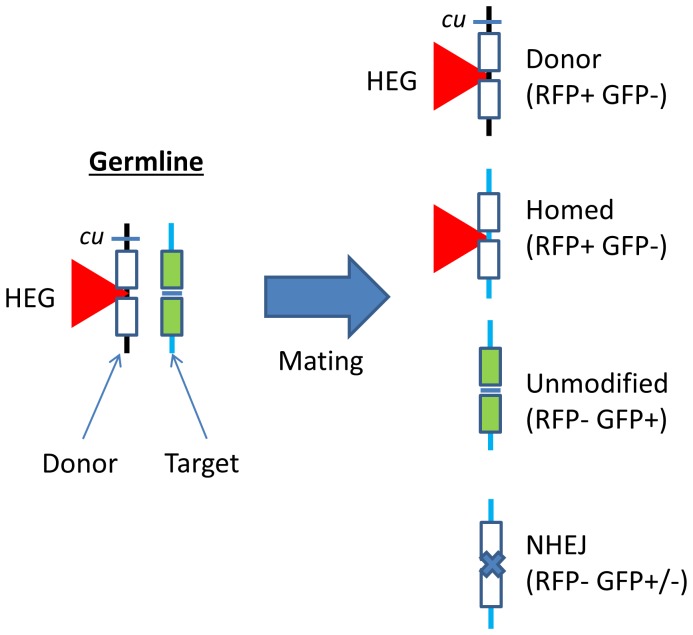
Homing assay. In this assay, donor and target constructs were placed at the same φC31 insertion site on homologous chromosomes (the donor and target chromosomes marked black and blue respectively). The target construct contains a GFP open reading frame (ORF) driven by an eye-specific promoter where the GFP ORF is split with an in-frame homing endonuclease recognition site (represented by adjacent green boxes). Transgenics bearing an intact target construct therefore exhibit GFP fluorescence in the eye. The donor construct has a homing endonuclease transcription unit is inserted into the HEG recognition site disrupting the GFP ORF and abolishing GFP fluorescence in the eye (loss of fluorescence represented by the GFP ORF being filled in white). Most constructs also include an RFP marker to allow the HEG insert to be tracked. Expression of the HEG in the germline causes cleavage of its recognition site in the target construct and subsequent repair leads to a number of different outcomes that can be differentiated by fluorescence and phenotypic markers as shown in the figure. The donor and target chromosomes are distinguished either with the linked *cu* marker (applicable with males only because of recombination) or a very closely linked mini-white marker within the donor construct (which is applicable to both sexes). It should be noted that NHEJ repair results in loss of GFP fluorescence in approximately two-thirds of cases only. The remaining third of NHEJ lesions can only be distinguished from unmodified targets by PCR and cleavage with I-*Sce*I.

Our homing assay has previously been described in detail [8]. A summary is shown in figure 1.

Both target and donor constructs were inserted into specific *attP* locations within the *Drosophila* genome using the φC31 integrase method such that they could be homologously juxtaposed *in vivo* [12], [13]. The donor and target constructs were differentiated by the use of linked chromosomal marker(s) (principally *cu*) and/or the presence of the eye colouration conferred by the presence of a functional *mini-white* marker on donor but not target constructs.

We elected to report the majority of results in terms of the directly observed metrics, GFP loss (fraction of all targets where GFP fluorescence is lost) and homed fraction (fraction of GFP-negative targets repaired *via* homologous recombination), using these as proxies for the fraction of total targets modified by DNA repair and the fraction of modified events attributable to homologous recombination (HR). A discrepancy arises between these measures because while HR invariably results in loss of GFP reporter fluorescence, non-homologous end-joining (NHEJ) repair can lead to in-frame lesions that are GFP-positive. This, in turn, results in the fraction of targets modified by repair being underestimated and the fraction of repaired targets arising from HR being correspondingly overestimated. In the case of pure NHEJ with in-frame events constituting a third of all repair events, the GFP loss would be a third lower than the true fraction of targets cleaved and repaired. While NHEJ in-frame lesions can be unambiguously identified by molecular biology, cost and labour constraints precluded its use with the large number of assays performed in this work. It is possible to estimate the number in-frame NHEJ events as a proportion of the number of out-of-frame NHEJ events but the accuracy of these estimates is doubtful. Since we are primarily interested in *comparing* related homing constructs, we reason that this caveat is of relatively low importance. When comparing the effect of 3′-UTRs on performance, the promoter and consequently the propensity to generate in-frame NHEJ events is unchanged and GFP loss is then a valid proxy for HEG activity. Similarly, when comparing the effect of genome location, the constructs are identical and NHEJ propensity remains fairly similar at the different locations. For constructs with radically different NHEJ propensities, e.g. when comparing *Rcd-1r-* and *Mst87F-*driven constructs, meaningful comparisons of HEG activity are impossible since precise religation dominates in the mitotic stages while double-strand break repair is greatly reduced at the later stages of spermatogenesis when NHEJ events appear to dominate [8], [14], [15]. A lower HEG activity is therefore required to generate a scorable repair lesion late in spermatogenesis than in the spermatogonial cells where HR occurs. Finally, the metric that of greatest import when comparing construct performance in HEG gene drive is the fraction of total targets homed which has the advantage of being directly measurable and immediately relevant.

To ensure comparability, all of the results in Table 1 were obtained with integrants at the *attP2* site [12]. Crosses unique to this work presented in this table are:

**Table 1:** Summary of results of various promoter/3′-UTR combinations for transgenes at *attP2*.

Promoter	3′-UTR	Construct	GFP loss	Homing fraction	Fraction of targets homed
*βTub85D*	*Hsp70Ab*	*βTub85D*-HEG-1	70% (688/985)^1^	1% (1/94)	<1%
*Mst87F*	*Hsp70Ab*	*Mst87F*-HEG-1	61% (638/1041)^1^	0% (0/94)	Nil
*Hsp70Ab*	*Hsp70Ab*	*Hsp70Ab-HEG1*	26% (78/296)^1^	78% (225/287)	20%
*bam*	*Hsp70Ab*	*bam*-HEG-1	0% (0/55)	ND^2^	ND^2^
	Native 3'-UTR	*bam*-HEG-2-*bam*	2.8% (66/2326)	64% (42/66)	1.8%
	*βTub56D*	*bam*-HEG-2*-βTub56D*	9.1% (357/3910)^1^	69% (245/357)	6.3%
*vas*	*Hsp70Ab*	*vas-HEG1*	1.9% (7/361)	ND^2^	ND^2^
	SV40 early	*vas*-HEG-2-*SV40*	11% (210/1873)	52% (110/210)	5.9%
	Native 3'-UTR	*vas*-HEG-2-*vas*	36% (328/911)	48% (157/328)	17%
	*βTub56D*	*vas*-HEG-2*-βTub56D*	33% (1234/3764)^1^	42% (523/1234)	14%
*Act5C-P (males)*	*Hsp70Ab*	*Act5C-P*-HEG-1	5.5% (44/793)	34% (15/44)	1.9%
	*βTub56D*	*Act5C-P*-HEG-2*-βTub56D*	53% (671/1272)^1^	38% (252/671)	20%
*Act5C-P (males),* 18°C	*βTub56D*	*Act5C-P*-HEG-2*-βTub56D*	19.0% (609/3198)	31.5% (192/609)	6.0%
*aly*	*Hsp70Ab*	*aly-HEG-1*	38% (417/1094)^1^	2% (2/94)	1.8%
	*βTub56D*	*aly*-HEG-2*-βTub56D*	70% (754/1083)	6.2% (47/754)	4.3%
*bgcn*	*Hsp70Ab*	*bgcn-*HEG-1	58% (676/1162)	0.3% (1/282)	∼0.1%
	Native 3'-UTR	*bgcn*-HEG-2-*bgcn*	62% (1486/2385)	0.3% (4/1486)	0.2%
	*βTub56D*	*bgcn*-HEG-2*-βTub56D*	65% (642/992)	0.5% (3/642)	0.3%
*Rcd-1r*	*βTub56D*	*Rcd-1r*-HEG-2*-βTub56D*	37% (1273/3422)	61% (782/1273)	23%
*Rcd-1r*, 18°C	*βTub56D*	*Rcd-1r*-HEG-2*-βTub56D*	15% (395/2614)	38% (152/395)	5.8%
*CG9576*	*βTub56D*	*CG9576*-HEG-2*-βTub56D*	14% (232/1612)	12% (27/232)	1.7%

1 previously reported in [8].

2 ND: not done.

♂ *w*;; *attP2*{*bam*-HEG-1} *cu*/*attP2*{pDarkLime} x ♀ *y w*;;*attP2 cu*

♂ *w*;; *attP2*{*bam*-HEG-2-*bam*} *cu*/*attP2*{pDarkLime} x ♀ *y w*;;*attP2 cu*

♂ *w*;; *attP2*{*vas*-HEG-1} *cu*/*attP2*{pDarkLime} x ♀ *y w*;;*attP2 cu*

♂ *w*;; *attP2*{*vas*-HEG-2-*SV40*} *cu*/*attP2*{pDarkLime} x ♀ *y w*;;*attP2 cu*

♂ *w*;; *attP2*{*vas*-HEG-2-*vas*} *cu*/*attP2*{pDarkLime} x ♀ *y w*;;*attP2 cu*

♂ *w*;; *attP2*{*Act5C-P*-HEG-1} *cu*/*attP2*{pDarkLime} x ♀ *y w*;;*attP2 cu*

♂ *w*;; *attP2*{*aly*-HEG-2*-βTub56D* } *cu*/*attP2*{pDarkLime} x ♀ *y w*;;*attP2 cu*

♂ *w*;; *attP2*{*bgcn*-HEG-1} *cu*/*attP2*{pDarkLime} x ♀ *y w*;;*attP2 cu*

♂ *w*;; *attP2*{*bgcn*-HEG-2-*bgcn*} *cu*/*attP2*{wDarkLime} x ♀ *y w*;;*attP2 cu*

♂ *w*;; *attP2*{*bgcn*-HEG-2*-βTub56D* } *cu*/*attP2*{wDarkLime} x ♀ *y w*;;*attP2 cu*

♂ *w*;; *attP2*{*Rcd-1r*-HEG-2*-βTub56D*} *cu*/*attP2*{wDarkLime} x ♀ y *w*;;*attP2 cu*

♂ *w*;; *attP2*{*CG9576*-HEG-2*-βTub56D*} *cu*/*attP2*{wDarkLime} x ♀ y *w*;;*attP2 cu*

The twin transgene cross presented in Table 2 was:

**Table 2:** Homing performance is expression-limited.

Transgene copy number	GFP loss	Home/GFP-	Fraction of targets homed
1 copy^1^	37% (1273/3422)	61% (782/1273)	23%
2 copies^2^	77% (901/1170)	71% (636/901)	54%

1 *attP2{Rcd-1r-HEG-2-βTub56D }.*

2 *attP40{ Rcd-1r-HEG-2-βTub56D }/+; attP2{Rcd-1r-HEG-2-βTub56D}/attP2{wDarkLime}.*

♂ w;attP40 { Rcd-1r-HEG-2-βTub56D }; attP2{Rcd-1r-HEG-2-βTub56D} cu/attP2{wDarkLime} x ♀ y w;;attP2 cu

Additional crosses performed for Table 3 were:

**Table 3:** Genome location and *Rcd-1r*-HEG-2*-βTub56D* transgene performance.

Chromosomal band	GFP loss	Homing (as fraction ofGFP-negative targets)	Homing (as fraction of all targets)	Nearest genes
*2L; 25C6 (attP40)*	39% (1467/3733)	58% (846/1467)	23%	Msp-300: ubiquitous
*2R; 51D (attP51D)*	53% (1541/2884)	71% (1094/1541)	38%	CR43622, CG33467:male-specific
*3L; 68A4 (attP2)* 1	37% (1273/3422)	61% (782/1273)	23%	CG6310, Mocs: ubiquitous
*3R; 86Fb (attP86Fb)*	58% (2671/4573)	58% (1538/2671)	34%	Clc: ubiquitous

1 extracted from Table 1.

♂ *w*; *attP40*{*Rcd-1r*-HEG-2*-βTub56D*}/*attP40*{wDarkLime} x ♀ *y w*;;*attP2 cu*

♂ *w*; *attP51D*{*Rcd-1r*-HEG-2*-βTub56D*}/*attP51D*{wDarkLime} x ♀ *y w*;;*attP2 cu*

♂ *w*;; *attP86Fb* {*Rcd-1r*-HEG-2*-βTub56D*} *cu*/*attP86Fb* {wDarkLime} x ♀ *y w*;;attP2 *cu*

The additional cross in Table 4 is:

**Table 4:** Co-expression of *Rcd-1r*-HEG*-βTub56D* and *bgcn*-HEG*-βTub56D*.

Construct	GFP-negative eventsarising from HR^1^	GFP-negative eventsarising from NHEJ^1^	GFP-positive^1^
*attP40*{Rcd-1r-HEG-2*-βTub56D* 2	23% (846/3733)	17% (621/3733)	61% (2266/3733)
*attP2*{*bgcn*-HEG-2*-βTub56D*}^3^	0.3% (3/992)	64% (639/992)	35% (350/992)
Both	27% (296/1082)	47% (507/1082)	26% (279/1082)

1 as fraction of all targets.

2 restated from Table 3.

3 restated from Table 1.

♂ *w*; *attP40*{*Rcd-1r*-HEG-2*-βTub56D*}; *attP2*{*bgcn*-HEG-2*-βTub56D* } *cu*/*attP2*{wDarkLime} x ♀ *w*;;*attP2 cu*

All crosses were performed at 25°C unless otherwise stated.

The ectopic homing assay in Table 5 was performed with single male crosses of type:

**Table 5:** Ectopic homing.

Construct	Target class	Donor	Acceptor	GFP loss^1^	Homed events
*vas*-HEG-2*-βTub56D* 2	Unpaired	*attP1* (55C4)	*attP2* (68A4)	29% (302/1057)	5
*Act5C-P*-HEG-2*-βTub56D* 2	Unpaired	*attP1* (55C4)	*attP2* (68A4)	55% (181/327)	3
*Rcd-1r*-HEG-2*-βTub56D*	Unpaired	*attP40* (25C6)	*attP2* (68A4)	67% (1810/2685)	20
*Rcd-1r*-HEG-2*-βTub56D*	Paired	*attP40* (25C6)	*attP2* (68A4)	21% (628/3011)	21 (10 of 41 crosses)

1 as fraction of all targets. Figures for actual GFP-negative and total target counts follows.

2 previously reported in [8] and included here for ease of comparison.

♂ *w*; *attP40*{*Rcd-1r*-HEG-2*-βTub56D*}/+; *attP2*{wDarkLime} x ♀ *w[1118]*

♂ *w*;; *attP2*{wDarkLime} x ♀ *w*; *attP40* {*Rcd-1r*-HEG-2*-βTub56D*}

♂ *w*;; *attP2*{wDarkLime} x ♀*w*; *attP40*{*Rcd-1r*-HEG-2*-βTub56D*}/+; *attP2*{wDarkLime}/+

Flies of the genotype *w*; *attP40*{*Rcd-1r*-HEG-2*-βTub56D*}/+; *attP2*{wDarkLime} were identified by increased GFP fluorescence as a result of homozygosity for wDarkLime. It was necessary to transmit the wDarkLime insertion *via* the female line to avoid HEG expression mutating the I-*Sce*I GFP target prior to creating the transheterozygote. Scoring for increased GFP fluorescence was a difficult procedure and potentially error-prone. To control for this, the transheterozygotes were evaluated in single male crosses so those involving a transheterozygote hemizygous for wDarkLime could be readily distinguished by an anomalously high proportion of GFP-negative progeny (>50% GFP loss). The observed GFP losses for each of the 42 crosses showed all but one result yielding GFP losses scattered around the average GFP loss of 21% with one well-separated outlier at 60%. That outlier was excluded from further analysis.

### Fluorescence Microscopy

Flies were scored for their fluorescence status with a MZ16F microscope (Leica) using the GFP2 and TXR filter sets.

### *in situ* Hybridisation

I-*Sce*I transcripts were detected with a PCR-generated anti-sense probe against a part of the I-*Sce*I coding region using the protocol described in [16]. A sense probe to the same region was used as control. Details of this probeset were previously published in [8].

## Results

### The 3′-UTR Strongly Influences Level of HEG Expression

Our original HEG-1-based constructs used the *Hsp70Ab* 3′-UTR derived from the *70I-SceI* construct [8], [17]. We observed that while this vector yielded high levels of HEG activity with promoters expressing later in spermatogenesis (e.g. *aly, βTub85D, Mst87F*), there was little or no activity when used with promoters targeted to the early germline stem cell and spermatogonial stages (i.e. *bam, vas*) (see Table 1). Other 3′-UTRs were therefore investigated as a means of improving expression.

The *vas* promoter was chosen because it has small but detectable homing activity with the *Hsp70Ab* 3′-UTR and it was coupled to several other 3′-UTRs to investigate the impact of 3′-UTR choice on testis HEG activity. These UTRs included the SV40 early intron/3′-UTR combination deployed in the extensively-used pUAST vectors [18], the *vas* native 3′-UTR and the *βTub56D* 3′-UTR. The first was selected because it contained an intron and splicing has previously been reported to be required for strong transgene expression in *Drosophila* [19]. The latter was chosen because *βTub56D* is known to be expressed at high levels at the early stages of spermatogenesis [20].

From Table 1, it is evident that when coupled with promoters active during early stages of spermatogenesis (*bam*, *vas*, *Act5C-P*), the *Hsp70Ab* 3′-UTR performed particularly poorly in comparison to the other 3′-UTRs (*Hsp70Ab*<<SV40< *vas* ≈ *βTub56D*). In contrast, for promoters driving expression during later stages *(aly*, *bgcn*), the *Hsp70Ab* 3′-UTR-mediated activity was only modestly reduced with the *aly* promoter and approached the *βTub56D* 3′-UTR in performance with the *bgcn* promoter. While the 3′-UTRs of genes known to be expressed in the testis performed equally well, it was surprising that the popular SV40 early intron/3′-UTR combination only yielded HEG activity at 30% of that observed with the former 3′-UTRs. Since the results showed no notable advantage in using *vas* native 3′-UTR, we based our subsequent HEG-2 design around the *βTub56D* 3′-UTR [8].

We also investigated whether native 3′-UTRs raised expression. The original *bam* promoter-driven transgene with the *Hsp70Ab* 3′-UTR had negligible HEG activity. When it was coupled with the *bam* 3′-UTR HEG activity, as expressed by the loss of GFP at the target site, rose to ∼3% but this was considerably lower than the 9% achieved with the *βTub56D* 3′-UTR without appreciable change in homing efficiency (Table 1). In the ovary, RBP9 acts to downregulate *bam* transcripts *via* sites within the *bam* 3′-UTR [21]. It is possible that the reduced expression with the *bam* 3′-UTR may also arise from this mechanism: according to the Spermpress microarray data *RBP9* is expressed in the mitotic cell population of the testis (see below) [22].

### Identification of Promoters that can Mediate HEG Drive Efficiently

We previously reported that efficient homing in the testis requires HEG expression at the spermatogonial stage [8]. Although large increases in HEG activity as evidenced by GFP loss were secured by the use of the *βTub56D* 3′-UTR, the highest levels of HEG activity did not correlate with similarly high rates of homing (Table 1). Promoters that had the potential to raise spermatogonial expression further were therefore sought.

Genetic evidence indicates that *bgcn* is functional in the germline stem cell and during spermatogonial stages of spermatogenesis [23]. While previous workers fused the 2 kb upstream of the transcription start site to a *bgcn* cDNA/GFP fusion to achieve expression and phenotypic rescue, such a fragment would have extended deep into the neighbouring *TBPH* coding region [24]. Instead, we used a 817 bp fragment as the promoter sequence since that extended upstream from the *bgcn* start codon and included all intergenic space between *bgcn* and *TBPH* as well as the entire 3′-UTR of *TBPH*. However, the homing results we obtained were contrary to our expectations. Although high levels of HEG activity were achieved with the *Hsp70Ab* 3′-UTR, NHEJ dominated, suggesting that the promoter was not driving in the spermatogonial cells. We considered the possibility that the *Hsp70Ab* 3′-UTR suppressed earlier expression and investigated the effect of using the native *bgcn* 3′-UTR and the *βTub56* 3′-UTR. Although we achieved a further increase in HEG activity with these 3′-UTRs, homing activity remained extremely low.

We therefore sought further promoters with the desired pattern of expression and undertook a bioinformatics search of the Spermpress microarray data [22]. Vibranovski *et al* dissected the testis into three regions termed the mitotic, meiotic and post-meiotic zones and performed microarray expression analysis on each region. A gene like *bam* that expressed solely in spermatogonia should show declining expression during progression through spermatogenesis and that was observed in the Spermpress data. We therefore sought genes that were expressed more strongly than *bam* but showed the same temporal expression profile, while recognising that the Spermpress categories only approximate the biological categories of spermatogonia/spermatocytes/spermatids. Further, a search of the tissue specific gene expression data was performed to restrict the candidates to those that were only expressed in the adult gonad as reported in FlyAtlas [25]. Two candidates were examined further: *Rcd-1r* (CG9573) and CG9576 (Table 1). Of these, only the *Rcd-1r* promoter showed significant HEG activity *and* homing in our assay (Table 1).

**Figure 2:**
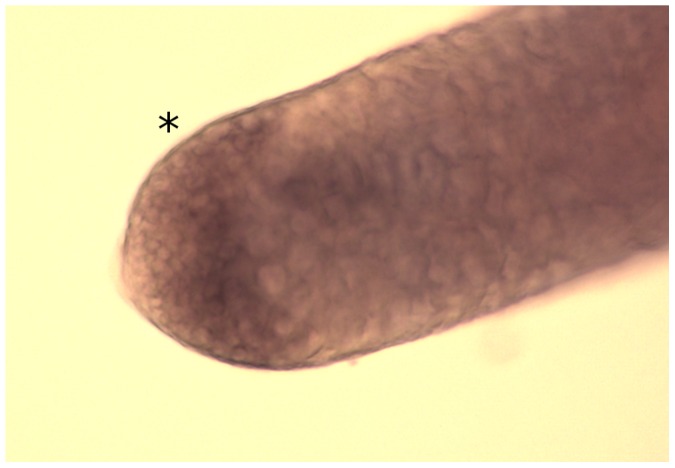
*in situ* hybridisation of I-*Sce*I expression *in Rcd-1r*-driven transgenics. The I-*Sce*I transcript is clearly detected in the spermatogonial population of the testis (marked by adjacent black asterisk). *In situ* hybridisation was performed as described in [8].

The *Rcd-1r* promoter resulted in fourfold more HEG activity (37%) than the *bam* promoter (9%) that it was intended to replace. Although GFP loss achieved with *Rcd-1r* (37%) was lower than that of the Act5C promoter/P-intron combination (Act5C-P; 53%), it combined with a much higher homing fraction (61% vs 38%) resulting in a comparable fraction of total target chromosomes repaired *via* homing. *in situ* hybridisation showed that the *Rcd-1r* promoter-driven transgene expressed specifically in spermatogonia (see figure 2). This promoter, in combination with the *βTub56* 3′-UTR, was therefore chosen for our subsequent constructs.

### Performance is Expression-limited

The fraction of total targets repaired as homing events is dependent on both the fraction of targets cut by the HEG and the homing fraction, that is, the proportion of events repaired via homologous recombination. It was therefore surprising to find that the fraction of total targets homed with the *Act5C*-P promoter (20%) and previously reported results with the *Rcd-1r* promoter (23%) and the *Hsp70* promoter (21%) were all very close to each other, which might suggest an inherent biological limitation to homing in *Drosophila*. We therefore investigated whether higher levels of I-*Sce*I expression could raise the fraction of total targets homed. To achieve this, we supplied a further copy of the *Rcd-1r*-driven transgene on chromosome 2 in addition to that on chromosome 3. The fraction of total targets homed doubled, suggesting that the homing was limited by expression of the HEG rather than any intrinsic biological limitation (Table 2).

### Genome Context Influences Homing *via* Level of HEG Expression

Genome context could have a role in determining the propensity for homologous recombination and to investigate this, we examined the performance of homing at three additional autosomal *attP* locations (Table 3) [13]. GFP loss varied between approximately 39% (*attP40*) to 58% (*attP86Fb*) at different sites while the homing fraction varied from approximately 58% to 71%, combining to yield a roughly two-fold variation in fraction of total targets homed, ranging from approximately 23% to 38%. The bulk of observed variation in the fraction of total targets homed appears to have arisen from genome context effects on HEG expression leading to variation in HEG activity, rather than from changes in the homing efficiency. This is in line with previous work reporting that the same transgene integrated at different *attP* integration sites can result in widely varying expression in a tissue-dependent manner [26]. Since the fraction of total targets homed at some of the alternative *attP* sites we assayed was significantly higher than the best achieved level at our original *attP2* inserts, it provided further evidence that homing performance in *Drosophila* scales with the level of HEG expression.

### *bgcn* Promoter Expression Occurs after the Spermatogonial Stage

The anomalous results obtained with our version of the *bgcn* promoter could potentially be attributed to expression in germline stem cells (GSCs) that are refractory to HR. Since the GSC stage precedes the HR-responsive spermatogonial stage during differentiation, one would expect that when I-*Sce*I is simultaneously supplied from an *Rcd-1r*-driven transgene and a *bgcn*-driven transgene, NHEJ should dominate the observed repair events. Conversely, if the NHEJ events due to HEG activity driven by the *bgcn* promoter arose from expression subsequent to the spermatogonial stage, the majority of repair events should be attributable to HR. When tested experimentally using an *Rcd-1r* driven HEG at the second chromosome *attP40* site and measuring homing between the *bgcn* driven HEG and its target at the third chromosome *attP2* site, homing rates were very similar to those observed with *Rcd-1r* driven HEG alone at *attP40.* We suggest the difference is due to targets that were unmodified with *Rcd-1r*-driven HEG expression subsequently being almost wholly converted by NHEJ events by the presence of *bgcn*-driven HEG expression (Table 4). Thus we conclude that the *bgcn* promoter fragment we used only drives strong post-spermatogonial expression.

### The I-*Sce*I Nickase does not Mediate Homing in the Testis

Previous work suggested that, like double strand breaks, single-strand nicks could also induce HR [27], [28]. To test this, an *Rcd-1r* promoter-driven transgenic stock, *attP2*{*Rcd-1r*-K227M*-βTub56D*}, was generated using the K227M variant of I-*Sce*I that has previously been shown to have a strong preference for nicking the target on a specific strand [29]. In all other respects, the construct used was was identical to its progenitor, *attP2*{*Rcd-1r*-HEG-2*-βTub56D*}. No HR or NHEJ events were observed in our assay after scoring 418 target chromosomes, suggesting that this variant is not active in our assay or that the single strand breaks are not recombinogenic in our HEG assay.

### HEGs can Home to Paired Ectopic Sites

Rather than employ homologous recombination to insert a HEG construct at its correct target location in the genome, which may be technically challenging with some species, ectopic homing may be used to move a randomly integrated HEG donor construct to its recognition site in the genome. Our previous work established that homing between a donor HEG construct at the *attP1* site on chromosome 2 to a target construct at the *attP2* site on chromosome 3 occurred frequently enough that one could be confident that an ectopic jump could be isolated by screening several thousand progeny [8]. However, that experiment only employed a hemizygous target construct at the *attP2* site: in the natural configuration, a pair of targets would be present at cognate sites on the homologous chromosomes. HR could potentially favour the use of homologous template to the extent that ectopic homing becomes an exceedingly rare outcome. To allay this concern, we performed an ectopic homing experiment using both paired and unpaired wDarkLime targets at *attP2* and a *Rcd-1r*-HEG*-βTub56D* donor at *attP40* (Table 5). Surprisingly, we found that the frequency of ectopic homing in the paired case was of a similar magnitude to that observed in the unpaired case. The modest number of ectopic jumps observed is too low to precisely determine the ectopic homing rate but is adequate to demonstrate that ectopic jumps can readily be identified on screening an acceptable number of progeny.

### Low temperature Reduces HEG Spread

As the natural environment within which HEG gene drive is required to act is subject to diurnal and seasonal changes, we also determined the effect of ambient temperature on homing performance. When the *attP2*{*Rcd-1r*-HEG-2*-βTub56D*} cross was performed at 18°C rather than the usual 25°C, both HEG activity (as evidenced by loss of GFP fluorescence) and the efficiency of homing fell sharply, with the fraction of total targets homed declining nearly fourfold. We repeated this experiment with a different transgenic, *attP2*{ *Act5C-P*-HEG-2*-βTub56D*}, which also showed declines in both HEG activity and homing efficiency leading to a very similar overall decline in the fraction of total targets homed. The data suggest ambient temperature could be an important determinant of HEG gene drive performance. (Table 1).

## Discussion

Previous studies establishing the feasibility of HEG-based gene drive in Diptera indicated that the process was more efficient in *Anopheles gambiae* than in *Drosophila melanogaster.* In this study, we have shown that homing in *Drosophila* scales with the level of HEG expression raising the question of whether poor performance relative to *Anopheles* is solely due to lower expression or is also affected by other constraints. First, unlike *Anopheles*, *Drosophila* spermatogenesis proceeds *via* an achiasmate mechanism and crossovers are absent from the male germline. We speculate that this has the effect of restricting homologous recombination to the transit-amplifying mitotic spermatogonial stage. In contrast, with *Anopheles*, HR may still be operational during the early spermatocyte stage prior to the first meiotic division since it mediates crossover events in the germline. The longer period during which HR is available is expected to allow higher rates of homing for a given level of HEG activity.

We have also shown that optimisation of promoter and 3′-UTR choice can raise HEG activity levels very substantially. We were initially surprised that 3′-UTR choice had such a pronounced effect in our homing assays. Even though the *Hsp70* 3′-UTR has been used in a number of *Drosophila* constructs, there is experimental evidence that it contributes toward Hsp70 induction by destabilising its mRNA in the absence of heat stress [30]. However, mRNA destabilisation does not fully explain our observations: our previously described *Hsp26* promoter-driven HEG construct achieved modest levels of homing (11% homing fraction) when combined with the *Hsp70Ab* 3′-UTR under unstressed conditions. One may speculate that this promoter may have the ability to override the destabilising effect of the *Hsp70Ab* 3′-UTR, perhaps through stabilising factors bound to the polymerase complex being transferred to the nascent transcript. We also observed that the widely-used SV40 early intron/3′-UTR sequence performed poorly in our assays, consequently its use in constructs where high levels of expression during early stages of spermatogenesis is not recommended. Our current study suggests that the *βTub56D* 3′-UTR is able to support robust expression in the male germline.

We identified and tested the promoter region from *CG9573* as a potentially suitable spermatogonial promoter prior to the identification of the gene as *Rcd-1r* [31]. *Drosophila melanogaster* has three paralogues of *Rcd-1r*, related to a regulator of differentiation in yeast. We speculate that *Rcd-1r* is a regulator of spermatogonial differentiation and is expected to have a male-sterile phenotype. Indeed, the nearest male-sterile, *ms(2)29F*, was originally associated with a P-element insertion, *Rcd-1r* ^07717^, however this association has since been excluded and the location of *Rcd-1r*, within 3′UTR region of the overlapping gene *CG13102* makes further study of the gene difficult [31].

We were surprised that the *bgcn* promoter constructs resulted in very low levels of HEG activity during early stages of spermatogenesis but high activity during post-spermatogonial stages. We believe this is most likely due to the loss of distal control elements in the truncated promoter fragment we used or be due to an unanticipated interaction between the *bgcn* control elements and other elements within our constructs. Since our *bgcn* fragment includes the entire region between *bgcn* and its upstream neighbour, *TBPH*, as well as the entire *bgcn* 5′-UTR, we expected it would contain all key regulatory sites. *bgcn* intronic enhancers can be excluded since a genomic fragment containing the *bgcn* 5′ region and a substantial portion of the 3′ end of the adjacent *TBPH* gene was able to drive a *bgcn* cDNA to rescue *bgcn* mutants [17]. Therefore if the apparent low expression level of our *bgcn* driven HEG is the result of loss of regulatory elements necessary for gonial cell expression, these elements must reside within the *TBPH* gene or within the *bgcn* coding region. We note that evidence has been recently advanced to suggest that exonic enhancers are not uncommon [32]. In addition, if post-spermatogonial expression is a natural feature of the promoter, it suggests that *bgcn* may have further uncharacterised roles during later stages of spermatogenesis that are currently masked by its mutant phenotype at the spermatogonial stage.

The absence of homing associated with the expression of the I-*Sce*I nicking mutant is consistent with previous work showing that HR occurs less frequently when induced by nicks rather than DSBs, presumably because nick repair is rapid [27], [28], [33], [34]. The reduction was particularly pronounced when insertions are desired and homing unavoidably requires a large insert in the template [27]. In our experiment, we would expect approximately 96 homing events from the 418 chromosomes surveyed if wild type I-*Sce*I were used; the absence of any homing with the I-*Sce*I nickase suggests that nicks are at least two orders of magnitude less efficient in inducing HR in the *Drosophila* testis. While nickases do have the advantage of much lower NHEJ rates, and with that potentially slower development of HEG resistance due to the accumulation of NHEJ-induced sequence changes to the target site, the loss in homing activity is an excessive price to pay in the context of a HEG-based gene drive system.

The ability of an ectopic template to compete against a template at the homologous site was initially unexpected. However, since homing is restricted to fast-cycling transit-amplifying spermatogonia in these experiments, a large fraction of the genome will be in a post-replicative state regularly and sister chromatid repair is thereby enabled. Indeed, a large proportion of repair events may occur at this stage since HEG access to DNA, and consequently HEG cleavage, is restricted by chromosome condensation during M phase. From this perspective, an ectopic template will be frequently competing against a homologously-located template even in the unpaired case. This observation suggests that, at least in *Drosophilids,* it could be relatively easy to generate a stock with the correctly-homed transgene *via* normal transposon-mediated transgenesis followed by ectopic homing rather than requiring a sophisticated targeted insertion system that is unlikely to be easily accessible in non-laboratory pest species.

The reduced homing performance at low temperatures observed in our experiments could have arisen from any of a variety of causes, including lower enzyme activity, lower expression of the HEG or reduced propensity towards HR. However, the strong dependence of I-*Sce*I-driven homing activity on temperature suggests that the temperature-activity profile of deployed HEGs is a relevant factor when modelling HEG spread. Habitats where a HEG-based control strategy could be envisaged may exhibit significant seasonal temperature variation. Where a cold-sensitive HEG insert exerts a fitness cost, its population frequency may be adversely affected in an environment where, for example, the peak breeding season coincides with a wet, cool season.

It was observed that efficient HEG drive was readily achieved in *Anopheles gambiae* but rather less so in *Drosophila melanogaster*, and this variation in response may suggest that HEG drive is an insect control strategy applicable only to a limited number of species [7], [8]. It appears likely that the difference arises from achiasmy in *Drosophila* males: since crossovers are absent in this species, the HR machinery is no longer required during meiotic stages and homing is consequently restricted to earlier, transit amplifying cells [8]. Achiasmy is widespread in higher Diptera [35], an order to which many insect pests belong, and the utility of HEG drive will depend on it being usable even in these less favourable circumstances.

We have shown here that even a semi-refractory species such as *Drosophila* is not inherently inferior in its ability to support homing: with sufficient HEG activity in the correct cell type, high levels of homing can be achieved. We also demonstrate that appropriate HEG activity can be achieved with judicious choices of 3′-UTRs and promoters. Moreover, chromosomal sites do not appear to vary much in their ability to support homologous recombination: rather, they act indirectly by influencing the expression of the HEG transgenes. HEG drive could therefore be potentially extended to genes that exhibit repressive chromatin in the testis by the use of insulator elements in transgene constructs [26]. The combination of these measures could allow HEG drive to be applied even in the most recalcitrant species.
